# Sex/gender-specific association between emotion regulation strategies and eating disorders symptoms: a systematic review

**DOI:** 10.1186/s40337-025-01434-4

**Published:** 2025-10-31

**Authors:** Aleksandra Sobieska, Joanna Kolanska, Paula Blauciak, Magdalena Ciechanowska-Jagodzinska, Karolina Zarychta

**Affiliations:** Faculty of Psychology in Wroclaw, SWPS University, Ostrowskiego 30b, 53–238 Wroclaw, Poland

**Keywords:** Eating disorder symptoms, Emotion regulation strategies, Gender, Sex

## Abstract

**Objective:**

Emotion regulation strategies (ERS) play a role in the development and maintenance of eating disorder (ED) symptoms. Findings on their associations with sex/gender and sex/gender differences in ERS usage in terms of ED symptoms remain unclear. This review aims to summarize the current state of knowledge on the associations between ERS and ED symptoms in both men and women, and the role of sex/gender differences in these associations.

**Methods:**

APA PsycInfo, APA PsycArticles, Academic Search Ultimate, Health Source: Nursing/Academic Edition, MasterFILE Premier, MEDLINE databases for studies published up to September 26, 2024 were reviewed. Included studies quantitatively assessed at least one ERS and ED symptoms and involved adult male and female participants. Excluded were animal studies, non-original research, studies involving only children or adolescents, qualitative studies, and studies that measured emotion regulation only as a general construct. Risk of bias was assessed. After screening 5,394 articles (from an initial 9,627), 16 studies were included.

**Results:**

Findings from 16 studies (10,974 participants) suggest that both sex/gender and ERS are predictors of ED symptoms. However, data on sex/gender differences in the use of ERS are insufficient to draw consistent conclusions.

**Discussion:**

The findings highlight the need for future research involving sex/gender -balanced samples. Further studies should include comparative analyses and explore the potential mediating and moderating roles of both ERS and sex/gender in the development of ED symptoms.

**Supplementary Information:**

The online version contains supplementary material available at 10.1186/s40337-025-01434-4.

## Plain Language Abstract

Emotion regulation strategies are associated with eating disorder symptoms. However, it is still unclear whether men and women differ in how they use these strategies. This review included 16 studies with nearly 11,000 adult men and women. Each study assessed at least one specific emotion regulation strategy and symptoms of eating disorders. Findings suggest that both sex and gender and the use of certain emotion regulation strategies are related to eating disorder symptoms. However, there is not enough consistent evidence to determine whether men and women differ in their use of these strategies. More research comparing men and women in the context of eating disorders is needed. Future studies should directly compare groups and examine how emotion regulation strategies and sex or gender are related to eating disorder symptoms.

**Supplementary Information:**

The online version contains supplementary material available at 10.1186/s40337-025-01434-4.

## Introduction

Eating disorders (EDs) are the third most common group of psychiatric disorders that can lead to life-threatening health effects [1]. They are more common in women, however, in men, these disorders are associated with a higher crude mortality rate - likely due to barriers in seeking help, which consequently may result in men receiving support only when symptoms are already severe [2]. EDs have become an important and widely studied psychiatric disorders, especially concerning the symptoms or mechanisms of development and maintenance of their symptoms, although research in the field of EDs are conducted mainly or even exclusively among women [3–5]. There are differences between women and men in terms of symptoms of EDs with men being more likely to purge by excessive physical exercises than by restrictive dieting or using laxatives and vomiting, which are more common in women [5, 6]. Women and men differ not only in the manifestation of ED symptoms but also in the underlying mechanisms of the development and maintenance of these symptoms. One of such mechanisms is the usage of the emotion regulation strategies (ERS) [7].

ERS, defined as a processes of implementing a conscious or unconscious goal to initiate, halt, or otherwise adjust the course of an emotion [8] play an important role in the development and maintenance of many psychiatric disorders such as depression or anxiety disorder [9]. The most common typology of ERS includes six strategies, which are defined in Table 1.

**Table 1:** ERS conceptualization [8–11]

ERS	Definition
Reappraisal	changing how one thinks about or appraises a given situation
Problem-solving	consciously attempting to change a stressful situation or mitigate its consequences
Acceptance	a non-elaborative, non-judgmental, present-centered awareness in which thoughts, feelings, and sensations are accepted as they are
Suppression	response-focused modulation, which entails inhibiting or suppressing an emotional experience once it has been activated or solidified
Avoidance	attempts to deny, minimize, or push away thoughts, feelings, and bodily experiences in response to negative emotions, as well as efforts to escape from or prevent situations that evoke negative emotions
Rumination	thinking repetitively and passively about negative mood states or about the causes and consequences of negative mood

This typology often divides the strategies into adaptive and maladaptive ones. Adaptive ERS effectively help to manage and modify unpleasant emotions in a healthy way. In contrast, maladaptive ERS may provide temporary relief but can ultimately prolong negative emotional experiences over time. Adaptive strategies include acceptance, problem-solving, and reappraisal. Maladaptive strategies encompass rumination, suppression, and avoidance [12]. An alternative approach to emotion regulation strategies found in the literature does not explicitly differentiate between adaptive and maladaptive strategies, but instead focuses on *emotion regulation flexibility*, understood as an access to a diverse repertoire of strategies, and the adaptive ability to shift between them as needed. For example, according to Aldao et al., the maladaptiveness or adaptiveness of a given strategy may depend more on its contextual appropriateness than on the strategy itself [13].

ERS also play a role in development and maintenance of ED symptoms [10, 11]. In systematic review and meta-analysis assessing the differences in ERS between individuals diagnosed with anorexia nervosa (AN) and bulimia nervosa (BN), the patients with different ED diagnoses did not differ in the use of maladaptive ERS. However, AN patients reported greater difficulties in applying adaptive strategies, such as reappraisal and acceptance [14]. In turn, experimental data suggest that rumination may causally intensify negative affect in individuals with EDs, as well as amplify negative body-related cognitions [15]. Furthermore, findings from the meta-analysis by Leppanen and colleagues in 2022 indicated that reduced use of adaptive ERS, such as acceptance, and greater reliance on maladaptive ERS - including suppression and avoidance - are associated with restrictive eating [10]. While limited, available empirical evidence on the role of ERS suggests the need to incorporate these strategies into theoretical models of EDs [16].

Noteworthy, Nolen-Hoeksema and Aldao indicates the differences in ERS between men and women in terms of the type and number of strategies used [9]. They found that women engage in a broader range of emotion regulation strategies than men, including rumination, reappraisal, problem-solving, and acceptance. These results were observed even when controlling for depressive symptoms, due to the gender differences in their prevalence. The meta-analysis on EDs and emotional regulation difficulties among adolescents and young adults indicated that emotional regulation issues are more likely to be associated with EDs in women than in men [17]. Nevertheless, it is important to note that the studies included in this meta-analysis did not measure specific ERS, but an overall emotion regulation difficulties [17]. Moreover, in adolescence, ERS are still developing with a tendency to stabilize in adulthood [18]. Therefore, the results of this meta-analysis cannot be generalized to adult population, which should be addressed when examining ERS in the context of more established regulatory patterns and their associations with EDs. Some studies suggest that women and men differ also in terms of emotion regulation flexibility with women being more flexible than men, although there is no data about these discrepancies in the context of EDs [19]. The differences between men and women also pertain to the neural mechanisms underlying emotion regulation [20]. In comparison to women, men demonstrated reduced activation in prefrontal regions associated with reappraisal or greater reductions in the amygdala, which is linked to emotional responding [20, 21]. Taking the above into consideration, both sex and gender are important to measure in research as they capture different but interrelated aspects of both ERS and EDs. Sex usually refers to a person’s biological characteristics, whereas gender refers to socially constructed roles and norms [22]. Including both can provide a more comprehensive understanding of individual differences and social influences.

Most research concerning the role of ERS and EDs has been conducted with the participation of women or with small subgroups of men [10, 11]. Their findings suggest that ERS play a significant role in the onset and maintenance of EDs; however, the current understanding of the role of sex/gender still remains unclear. In order to plan and conduct future studies in this area, it is crucial to precisely define the current state of knowledge and identify specific areas for further investigation. Therefore, the purpose of this review is to synthesize empirical evidence for (1) the associations between ERS and ED symptoms among both men and women, and to (2) analyze the role of sex/gender in these associations. The presence of sex/gender differences could have meaningful implications for the planning of diagnostic and therapeutic interventions.

In order to achieve the objectives of the review, the following research questions have to be answered:

1. What are the associations between ERS and ED symptoms among both men and women?
2. What is the role of sex/gender in the associations between ERS and ED symptoms?

Following definitions of variables were adopted: emotion regulation strategy was conceptualized as a process of implementing a conscious or unconscious goal to initiate, halt, or otherwise adjust the course of an emotion [8]. The definitions of specific ERS are presented in Table 1. It is important to note that we use the term *sex/gender* rather than referring to *sex* or *gender* individually, in order to address the ambiguity in the reviewed studies—many of which measured only one of these constructs or did not clearly specify which was being assessed (see Table 3, column 2).

## Methods

The review was conducted and reported according to *JBI Manual for Evidence Synthesis* [23] and PRISMA 2020 statement [24]. The review protocol was registered with the PROSPERO database under the number CRD42024600248.

### Eligibility criteria

The research question was constructed by using PICO mnemonic (*Population*,* Intervention*,* Comparison*,* Outcome*) [23] and based on publication characteristics. Therefore, the inclusion criteria were also developed according to the PICO framework. Studies included in the review: (1) had to be original studies (2) published between the inception of the databases and September 2024 (3) in peer-reviewed English-language journals, (4) had to measure at least one ERS and EDs symptomatology, (5) had the variables measured quantitatively, (6) had to include both male and female participants (of any race, education, place of residence etc.) (7) who were at least 18 years of age.

To obtain relevant articles, the following studies were excluded: (1) animal studies, case studies, reviews, systematic reviews, meta-analyses, theoretical contributions, position papers, dissertations, protocols, conference materials, and book chapters, (2) studies including only children and adolescent participants and (3) only female or only male participants, (4) duplicates, (5) studies with variables measured only qualitatively, (6) studies assessing emotional regulation as an overall variable without measuring specific ERS. Since some studies use the term gender and others use sex when referring to sex/gender differences, both types were included to cover all relevant research.

### Search strategy and information sources

In order to find scientific articles that could answer the review questions, a query was prepared with a combination of two groups of keywords referring to main variables: (1) *emotion regulation strategy* and (2) *eating disorders symptoms*. The exact search string used for this review was: *(“eating disorder” OR “anorexia” OR “bulimia” OR “binge eating” OR “orthorexia” OR “muscle dysmorphia” OR “body dysmorphia” OR “bigorexia”) AND (“emotion regulation” OR “emotion dysregulation” OR “affect regulation” OR “affect dysregulation” OR “appraisal” OR “reappraisal” OR “suppression” OR “rumination” OR “avoidance” OR “problem solving” OR “problem coping” OR “acceptance”).* A systematic search for the potentially eligible studies was performed using the EBSCO platform including six databases of peer-reviewed journals (APA PsycInfo, APA PsycArticles, Academic Search Ultimate, Health Source: Nursing/Academic Edition, MasterFILE Premier, MEDLINE). Query generation was performed on September 26, 2024.

### Selection of studies

The initial search generated 9627 records, which were analyzed using Rayyan software for duplicate detection. 2263 articles were excluded based on the analysis using this automation tool, and another 1970 records excluded after manual duplicate review performed by AS and KZ. Each abstract (after duplicates removal) was screened by a pair of independent researchers (from a four-researchers team: AS, JK, PB, MCJ). None of the researchers had access or insight into the selection decisions made by others. The disagreements between reviewers regarding the eligibility of a publication for inclusion in the review were discussed until consensus was reached. When needed, a senior team member (KZ) helped adjudicate unresolved discrepancies. To ensure high consistency in screening, the team (AS, JK, PB, MCJ) conducted a calibration exercise [25] on a sample of 15 articles. Each reviewer evaluated whether the study fulfilled the inclusion criteria and, in cases where it did not, indicated the reason for its exclusion. The consistency of 92% was achieved for the decision to include or exclude an article and of 82% for reasons for rejecting the articles. The consistency rate for exclusion reasons was satisfactory, however, to improve it and reduce the subjectivity in reporting the reasons for exclusion, the prioritization and sequential exclusion approach was applied [26], especially when two or more exclusion criteria applied to a single article. If an article could be excluded based on a higher-ranking rejection reason, that reason was assigned, even if a lower-ranking reason might also apply. Therefore, researchers were rejecting the article from the review following graded reasons: (1) different language than English; (2) key analyses unrelated to study aims; (3) a different population; (4) a different type of study; (5) not a peer-review journal. If two independent researchers assigned a different reason for rejection, then the article was rejected based on the reason higher in the hierarchy.

As a result of screening one hundred eleven articles were assessed for eligibility. After a full text assessment which involved four coders checking whether each article met the specified inclusion criteria, sixteen articles were included in the review. The disagreements between the decisions of the two investigators were discussed until consensus was reached or resolved by a third independent investigator (KZ).

**Fig. 1:**
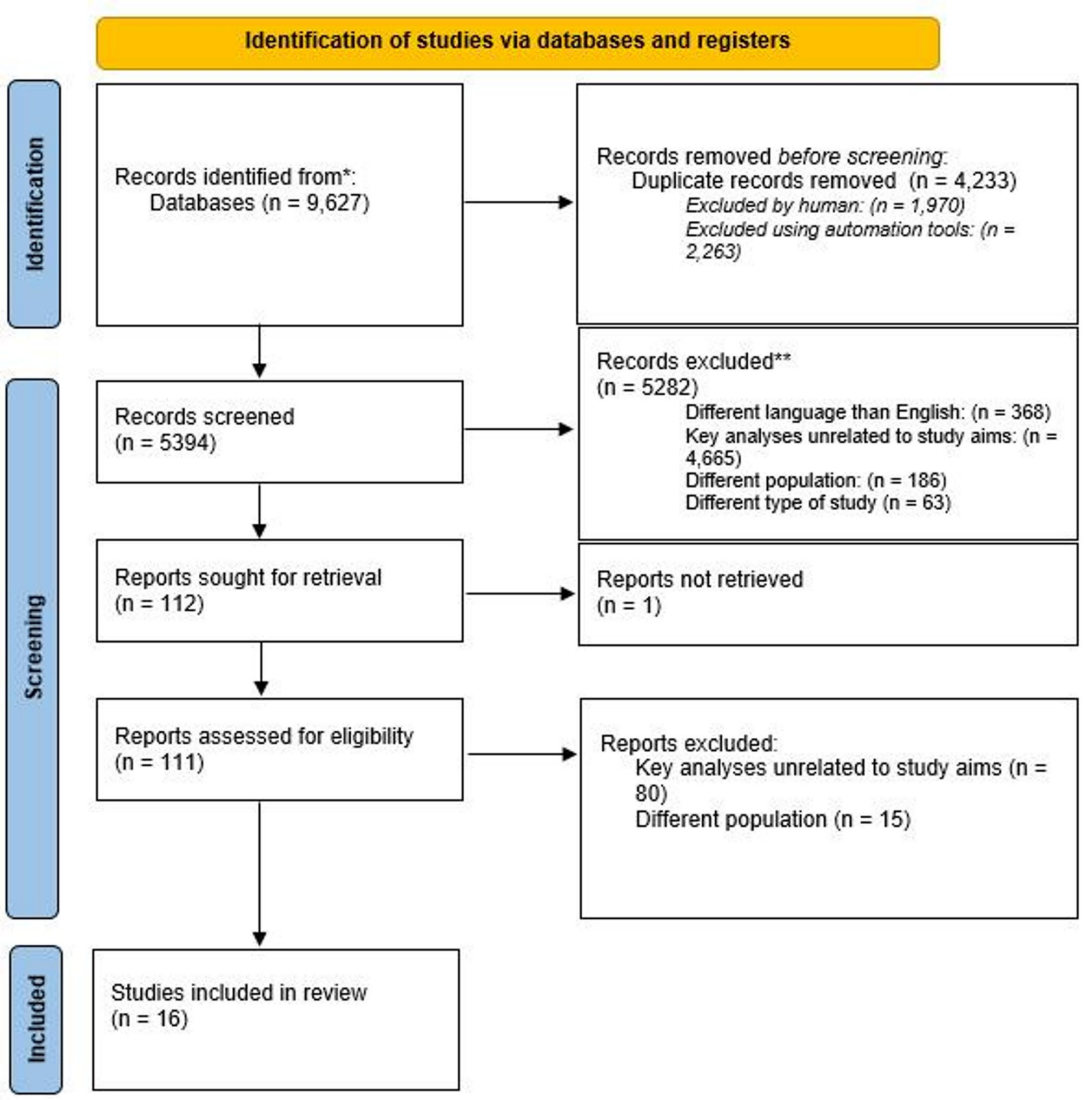
PRISMA flow chart of the performed systematic review

A detailed description of the subsequent stages of screening is presented on the PRISMA flow chart (Fig. 1).

### Data collection process

After sixteen articles were included in the final review, four independent researchers (AS, JK, PB, MCJ) performed data extraction. To address the study objectives, the following data was extracted from the included papers: (1) characteristics of the study population and sub-groups (sex/gender), (2) the type of the study, (3) the type of ERS assessed in the study, (4) the type of ED symptoms assessed in the study, and (5) the key results (lack or presence of the association, the type of association and sex/gender role). The data extracted from the included manuscripts, regarding study design, participant characteristics, measures, and findings related to the variables of interest were ultimately compiled into Table 3 and described in the Results section.

### Study risk of bias assessment

Two researchers (AS and KZ) rate the studies independently using the JBI critical appraisal checklist for analytical cross-sectional studies [23]. All included articles were evaluated along eight criteria. The overall risk of bias for the included studies was determined using the following cutoffs: (1) low risk of bias, if at least 70% of the assessed criteria were met; (2) moderate risk, if 50–69% of the criteria were met; and (3) high risk, if less than 50% of the criteria were met. Studies were rated as high quality and low risk of bias if they met 5 or more of the assessed criteria. The agreement between authors was 98%. No studies were excluded from the review after assessment of risk of bias. For details, see Table 2.

**Table 2:** *Risk of bias assessment of the included studies*

	JBI item										
Citation (Reference)	1. Were the criteria for inclusion in the sample clearly defined? (AS/KZ)	2. Were the study subjects and the setting described in detail? (AS/KZ)	3. Was the exposure measured in a valid and reliable way?	4. Were objective, standard criteria used for measurement of the condition? (AS/KZ)	5. Were confounding factors identified? (AS/KZ)	6. Were strategies to deal with confounding factors stated? (AS/KZ)	7. Were the outcomes measured in a valid and reliable way? (AS/KZ)	8. Was appropriate statistical analysis used? (AS/KZ)	Score (number of „YES”)		Overall appraisal (include/ exclude)
									AS/KZ	Final score	
Barnes & Tantleff-Dunn [30]	NO /NO	YES /YES	N/A	YES /YES	YES /YES	YES /YES	YES /YES	YES /YES	6/6	6	Include
Breithaupt et al. [38]	NO/NO	NO /NO	N/A	YES /YES	YES /YES	YES /YES	YES /YES	YES /YES	5/5	5	Include
Denning et al. [47]	YES /YES	YES /YES	N/A	YES /YES	YES /YES	YES /YES	YES /YES	YES /YES	7/7	7	Include
Eichler et al. [28]	YES /YES	YES /YES	N/A	YES /YES	YES /YES	YES /YES	YES /YES	YES /YES	7/7	7	Include
Gordon et al. [35]	NO /NO	NO /NO	N/A	YES /YES	YES /YES	YES /YES	YES /YES	YES /YES	5/5	5	Include
Jin et al. [45]	YES /YES	YES /YES	N/A	YES /YES	YES /YES	YES /YES	YES /YES	YES /YES	7/7	7	Include
Johnson et al. [36]	YES /YES	YES /YES	N/A	YES /YES	YES /YES	YES /YES	YES /YES	YES /YES	7/7	7	Include
Lavender et al. [43]	NO /NO	NO /NO	N/A	YES /YES	YES /YES	YES /YES	YES /YES	YES /YES	5/5	5	Include
McGarrity et al. [29]	YES /YES	YES /YES	N/A	YES /YES	YES /YES	YES /YES	YES /YES	YES /YES	7/7	7	Include
Mitchell et al. [44]	YES /YES	YES /YES	N/A	YES /YES	YES /YES	YES /YES	YES /YES	YES /YES	7/7	7	Include
Opwis et al. [31]	YES /YES	YES /YES	N/A	YES /YES	YES /YES	YES /YES	YES /YES	YES /YES	7/7	7	Include
Rawal et al. [27]	YES /YES	YES /YES	N/A	YES /YES	YES /YES	YES /YES	YES /YES	YES /YES	7/7	7	Include
Rosenbaum & White [42]	YES /YES	YES /YES	N/A	YES /YES	YES /YES	YES /YES	YES /YES	YES /YES	7/7	7	Include
Smith et al. [37]	NO /NO	YES /YES	N/A	YES/YES	YES/YES	YES/YES	NO/YES	YES/YES	5/6	5	Include
Turk et al. [39]	YES /YES	YES /YES	N/A	YES /YES	YES /YES	YES /YES	YES /YES	YES /YES	7/7	7	Include
Vuillier et al. [7]	NO/NO	YES /YES	N/A	YES /YES	YES /YES	YES /YES	YES /YES	YES /YES	6/6	6	Include

N/A - not applicable; final score - a score agreed between the two reviewers studies with scores ≥ 5 “YES” were considered of high quality and low bias, studies with scores ≤ 4 “YES” were considered of low quality and high bias

## Results

### Description of included studies

A total of *k* = 16 original studies were included in the review. The enrolled population consisted of *N* = 10,974 participants, of which 6972 (64%) of them were women, 3990 (36%) men and 12 gender diverse individuals. Study populations in individual studies differ significantly, ranging from 181 [27] to 2391 [28]. Ten studies were conducted among college students, one study among a non-clinical sample of same-gender twin pairs. Another five studies were conducted in community samples, twelve were conducted in both participants with and without ED symptoms. Among them, two studies concern patients with overweight or obesity. Given the aims of this review, we did not restrict our analysis to clinical populations to emphasize the relevance of subclinical symptomatology in understanding risk, maintenance, and prevention of full-threshold EDs. Given the aims, of the this review was not restricted to study and the fact that it did not specifically focus on clinical populations, and individuals presenting with eating disorder symptoms were not entirely excluded by the researchers, Therefore, studies that measured ED symptoms along a continuum of severity these articles were included in the review, provided they met the inclusion criteria *.* There was only one study conducted among a clinical EDs sample included in the review [29]. In three studies, individuals with certain types of EDs were excluded. Barnes and Tantleff-Dunn excluded individuals with symptoms of BN and AN but included participants with symptoms of BED [30]. In another study, Opwis et al. excluded individuals with clinically significant symptoms severity, such as engagement in purging behaviors, a Body Mass Index (BMI) below 18.5, and depression questionnaire scores indicating at least a moderate level of depressive symptoms [31]. Description of the included studies is presented in Table 3.

**Table 3:** Descriptive characteristics about included research

Citation (reference; study design)	Population (N, n, % gender, age, country)	Emotion regulation strategies and assessment (reliability coefficient)	ED symptoms and assessment (reliability coefficient)	Main findings of the research
Barnes & Tantleff-Dunn (cs) [30]	Patients with overweight or obesity (BMI ≥ 25) community sample with no current ED symptoms: *N* = 312, *n* = 213 women (68%), *n* = 99 men (sex), *M*_age_ = 40.00, *SD* = 12.15; USA	Suppression (the White Bear Suppression Inventory [WBSI] [40]; α = 0.92 (men), α = 0.94 (women)	Binge eating frequency and ED symptoms (the Eating Disorder Examination—Questionnaire [EDE-Q]; [78]) α = 0.70 to 0.87	Sex and BMI, together with suppression, significantly predicted ED symptoms, including restraint, eating concerns, shape concerns, weight concerns, and overall (global) ED symptoms. For binge eating frequency, only sex and BMI were significant predictors; suppression did not predict binge eating frequency
Breithaupt et al. (cs) [38]	College students:* N* = 353, *n* = 300 women (85%), *n* = 53 men (sex/gender); *M*_age=_ 21.93, *SD* = 5.78;, USA	Rumination (the Ruminative Response Scale [RRS] [32]; α = 0.86	BN symptoms (the bulimia and food occupation sub- scale from the Eating Attitudes Test [EAT-26] [66]) α = 0.79	Rumination predicted BN symptoms in both men and women as the effect of sex/gender was not significant
Denning et al. (cs) [47]	College students at risk for BPD symptoms: *N* = 181, *n* = 100 women (55.2%), *n* = 81 men (sex); *M*_age=_ 20.01, *SD* = 2.18; USA	Acceptance and avoidance (intrapersonal emotion regulation strategies subscales from the Difficulties in Interpersonal Regulation of Emotion questionnaire [DIRE] [46]) ω = 0.60 to 0.75	ED symptoms (based on the Eating Disorder Diagnostic Scale [EDDS] [79]) ω = 0.84	Neither acceptance nor avoidance predicted ED symptoms. Sex predicted ED symptoms
Eichler et al. (cs) [28]	Representative German sample: N = 2391 (53.4% women) (sex); *M*_age=_ 50.90, SD = 17.5; Germany	Reappraisal (subscale from the short form of the Emotion Regulation Questionnaire [ERQ-4] [28]; α = 0.81	ED symptoms (short form of the Eating Disorder Examination Questionnaire [EDE-Q8] [80]) α = 0.93	Lower levels of reappraisal linked to more ED symptoms. Women more often at risk to manifest lower levels of reappraisal than men
Gordon et al. (cs) [35]	College students: N = 780, n = 512 women (65.7%), n = 268 men (sex); *M*_age =_ 19.27, SD = 2.12; USA	Rumination (RRS [32]) α = 0.81 to 0.84	BED symptoms (the Binge Eating Scale [BES] [81]) α = 0.91	Brooding aspect of rumination and body dissatisfaction predicted BED symptoms with significant sex effect as women were found to be at higher risk for BED symptoms than men
Jin et al. (cs) [45]	College students: N = 1227, n = 892 women (72.7%), n = 323 men (26.3%), gender diverse n = 12 (0.1%) (gender); *M*_age_ = 20.52, SD = 3.05; USA	Reappraisal and suppression (the Emotion Regulation Questionnaire [ERQ] [41]) α = 0.76 to 0.85	ED symptoms (EDE‐Q 6.0 [82]) α = 0.96	Suppression and reappraisal mediated the link between insecure attachment and ED symptoms when gender being controlled for. Women and gender diverse subgroups reported higher ED symptoms than men
Johnson et al. (ts) [36]	Same-gender twin pairs: *N* = 744,* n* = 170 male twin pairs (87 monozygotic [MZ]; 83 dizygotic [DZ]), 14 singletons), *n* = 195 female twin pairs (107 MZ; 88 DZ and 7 singletons); M_age_ = 22.84, SD = 1.29; USA	Rumination (RRS [32]) and Rumination-Reflection Questionnaire [RRQ] [83]) α = 0.80 to 0.81 (RRS) *α* = 0.91 to 0.91 (RRQ)	ED symptoms (EDE‐Q 6.0 [82]) α = 0.94	Rumination associated with ED symptoms among female twins only mostly due to genetic/environmental factors. Women reported higher tendency to ruminate
Lavender et al. (cs) [43]	College students: *N* = 406, *n* = 187 women (46%), *n* = 219 men (gender); *M*_age_ = 19.1, *SD* = 1.5; USA	Suppression (WBSI [40]) α = 0.94	BN symptoms (Bulimia Test-Revised [BULIT-R] [84]) α = 0.93	Suppression predicted BN symptoms in both men and women
McGarrity et al. (cs) [29]	Patients with obesity undergoing pre-bariatric surgery psychological evaluation: *N* = 394, 75% women (sex/gender); *M*_age_ = 44.00, *SD* = 12.00; USA	Approach coping (including reappraisal and problem etc.) and avoidance coping (including avoidance, acceptance and resignation etc.) (Coping Responses Inventory [CRI] [48]) α = 0.80 to 0.87	BED symptoms (the Binge Eating Scale [BES] [81]) α = 0.88 ED symptoms (EDE-Q [78]) α = 0.67 to 0.84	Approach and avoidance coping predicted more BED symptoms and ED symptoms (eating, weight and shape concerns) in both men and women
Mitchell et al. (cs) [44]	Adults with self-reported EDs diagnosis and healthy adults: *N* = 841, n = 766 women (91.1%), n = 75 men (sex); M_age_ = 28.22, SD = 10.69; USA	Reappraisal and suppression (ERQ [41])	ED symptoms (EDE-Q [78])	Reappraisal predicted ED symptoms among BN and BED groups, but not AN and OSFED groups. Reappraisal were found to be a stronger predictor than the participants' gender
Opwis et al. (cs) [31]	Community sample with no active ED symptoms: N = 295, n = 205 women (69.5%), n = 90 men (gender); M_age_ = 30.39, SD = 8.99; Germany	Rumination (subscale from the Rumination-Suppression-8 Scale [RS-8] [34]) α = 0.89	BED and ED symptoms (EDE-Q [78]) α = 0.94	Rumination mediated the associations between gender and (1) subjective binge eating, (2) objective binge eating, (3) ED symptoms. Women reported higher tendency for both rumination and eating pathology
Rawal et al. (cs) [27]	College students: N = 177, n = 122 women (68.9%), n = 55 men (gender); M_age_ = 22.39, SD = 5.13; UK	Rumination (RRS [32]) Avoidance (the Acceptance and Action Questionnaire [AAQ] [49])	ED symptoms (EDE-Q [78] and the Eating Disorder Belief Questionnaire [EDBQ [85])	ED symptoms, and both brooding aspect of rumination and avoidance predicted EDs beliefs among both men and women
Rosenbaum & White (cs) [42]	Community sample: N = 436, n = 173 women (62%), n = 89 men (gender); M_age_ = 33.4, SD = 13.5; USA	Suppression (WBSI [40]) α = 0.95	BED symptoms (Eating Disorder Diagnostic Scale [EDDS] [79]) α = 0.76	Suppression mediated association between anxiety and BED symptoms for women, but not for men
Smith et al. (cs) [37]	College students: *N* = 263, *n* = 197 women (74.9%), *n* = 66 men (gender); M_age_ = 20.3, SD = 3.68; USA	Rumination (brooding subscale from RRS [32]) α = 0.87 Suppression (WBSI [40]) α = 0.93	ED symptoms (EDDS [79])	Brooding aspect of rumination was positively associated with binge eating in women, but not in men, and negatively with fasting in men, but not in women. Suppression was positively associated with vomiting frequency and fasting across genders, but for fasting the association was stronger for men than women
Turk et al. (cs) [39]	Community sample: *N* = 570, n = 369 women (64.7%), n = 201 men (gender); M_age_ = 29.78, SD = 9.70; UK	Rumination (Ruminative Thought Style Questionnaire [RTSQ] [33])	ED symptoms (EDE-Q; [78])	No significant indirect effects of self-compassion via rumination on ED symptoms were found for both men and women
Vuillier et al. (cs) [7]	Community sample with and without current ED symptoms: N = 1604, n = 973 women (60.7%), n = 631 men (gender); M_age_ = 26.66, SD = 11.17; UK	Reappraisal and suppression (ERQ [41]) α = 0.71 to 0.87 (men), α = 0.75 to 0.87 (women)	ED symptoms (EDE‐Q 6.0 [82]) α = 0.76 to 0.93 (men), α = 0.76 to 0.95 (women)	Suppression and reappraisal predicted ED symptoms (global index, restraint, and shape, weight, and eating concerns) with the effects stronger in women than men

*cs* cross-sectional design, *ts* twin study; in column 2: sex or gender indicates how authors referred to participants, sex/gender indicates that authors used these terms interchangeably

### Emotional regulation strategies and tools

#### Rumination

Rumination and its association with symptoms of eating disorders were assessed in seven of the analyzed studies. In five of these studies, researchers used the Ruminative Response Scale (RRS) as a measurement [32], while in the other two studies, the Ruminative Thought Style Questionnaire (RTSQ) [33] and a subscale from the Rumination-Suppression-8 Scale (RS-8) were employed as assessment tools (see Table 3) [34]. The results of the systematic review confirm a positive correlation between rumination and ED symptoms in both men and women, but particularly among females [31, 35–37].

Both ruminative tendencies and participants’ gender were identified as significant predictors of ED symptomatology in two of the included studies [31, 35]. Gordon and colleagues demonstrated that higher levels of brooding facet of rumination, along with being female, predicted concurrent BED symptoms [35]. The study also observed that BED symptoms were more prevalent among women than men. Opwis et al. reported that female gender was associated with a higher tendency for both rumination and eating pathology [31]. Notably, the level of rumination served as a significant mediator in the relationship between gender and various measures of disordered eating, including food cravings, BED symptoms, and overall ED symptoms. In three of the analyzed studies, only rumination was identified as a unique correlate of disordered eating symptoms, while participants’ gender did not play a significant role [36–38]. Breithaupt et al. found that higher levels of rumination predicted increased BN symptoms in both women and men, but particularly among those exhibiting lower levels of self-control [38]. In turn, Rawal et al. observed that greater concerns related to EDs predicted increased ruminative brooding, irrespective of gender [27]. The study conducted by Smith et al. revealed that the relation of rumination and ED symptoms varied by gender: high levels of ruminative brooding were associated with an increased frequency of binge eating in women but not in men [37]. Conversely, greater ruminative brooding in the male sample was linked to a lower frequency of fasting. The conclusion that the relationship between rumination and ED symptoms may differ by gender is further supported by a twin study conducted by Johnson et al. [36]. This research indicated that, in female twins, genetic factors explained a greater percentage of the covariance between ED symptoms and rumination compared to male twins, who also exhibited a lower tendency to ruminate. In one study, researchers also examined the potential mediating role of rumination in the relationship between self-compassion and overall ED symptoms, expecting differences between men and women [39]. However, the results indicated no significant mediating effect, nor any gender differences in the tendency to engage in ruminative coping.

#### Suppression

Suppression, as analyzed in seven publications, was assessed using two questionnaires: The White Bear Suppression Inventory (WBSI) [40] (four studies) and the Emotion Regulation Questionnaire (ERQ) [41] (three studies). Findings on suppression are inconsistent, although almost all indicate an important role of suppression in EDs. Barnes and Tantleff-Dunn demonstrated that women used suppression more frequently, but the strategy was employed by both women and men [30]. Rosenbaum and White reported that suppression was a significant predictor of ED symptoms in women, but not in men [42]. In turn, Lavender et al. also showed that suppression predicts bulimic symptoms in both women and men, with no gender differences [43]. In the study by Mitchell et al., suppression was a significant predictor for other specified feeding or eating disorder (OSFED), not for AN or BN [44]. Gender differences were not examined. In one study suppression was not a significant predictor of overall ED symptoms but was related to some specific symptoms; a significant positive correlation was found between suppression and vomiting frequency for both genders, as well as between suppression and fasting, with the latter effect notably stronger in males [37]. Findings from Jin et al. study did not directly compare women and men, as gender was controlled for due to differences in emotion regulation strategies and ED symptoms [45]. They found that suppression mediates the relationship between intolerance of uncertainty (IU) and ED. A mediating role of suppression between anxiety and binge eating disorder (BED), and only in women, was also observed in Rosenbaum and White’s study [42].

#### Avoidance

The avoidance strategy as a predictor of ED symptoms was tested in three cross-sectional studies, measured with three different scales: The Difficulties in Interpersonal Regulation of Emotion questionnaire (DIRE) [46] was applied in the study by Denning et al. [47], Coping Responses Inventory (CRI) [48] in the study by McGarrity et al. [29], and the Acceptance and Action Questionnaire (AAQ) [49] in the study by Rawal et al. [27]. As avoidance was included in only three studies, each employing a different measurement method, it is challenging to draw definitive conclusions. Nevertheless, the results support the hypothesis that this strategy is generally positively related to various symptoms of EDs in both men and women. Rawal et al. found that a higher level of ED concerns predicted the greater tendency to experiential avoidance, regardless of the participant gender [27]. Similar results were reported in the study conducted by McGarrity et al., which indicated that more avoiding was positively associated with more binge and disordered eating, as well as with concerns related to eating, weight, and shape [29]. These relationships were found to be independent of gender and BMI. In contrast to these findings, the research by Denning et al. demonstrated that the avoidance strategy was not a significant predictor of ED symptoms [47].

#### Reappraisal

Reappraisal strategy was assessed in five of the included studies, however in one study it was measured using the CRI as one of the components of the approach coping variable only, so it prevents drawing further conclusions regarding the relation between reappraisal and ED symptoms [29]. In the four remaining research, reappraisal was measured using the cognitive reappraisal subscale from Emotion Regulation Questionnaire (ERQ) [41] or its validated German short form (ERQ-4) [28]. In the study by Eichler et al., the emotion regulation model defined as “dysregulated” was associated with the lowest frequency of cognitive reappraisal use and the highest frequency of ED symptoms [28]. This model was also more prevalent among women than men. Moreover, another study found that lower tendency to use cognitive reappraisal strategy is a significant predictor of generalized ED symptoms, and this association was stronger in women than in men [7]. Mitchell et al. demonstrated that low inclination to use reappraisal strategy is a significant predictor of ED symptoms only in certain diagnoses, finding an association for BN and BED, but not for AN or OSFED [44]. Notably, in a sample of women and men with self-reported BED diagnoses, reappraisal level proved to be a better predictor of symptoms than the participants’ gender.

#### Acceptance

Acceptance was measured with DIRE [29] in one study, which indicated that it was not a significant predictor of EDs [47]. Acceptance was also considered a part of avoidance coping in the research by McGarrity et al., however, a conceptualization of acceptance was not provided by the authors, and thus, it is unclear whether it aligns with the definition of acceptance assumed in this review [29].

#### Problem-solving

None of the included studies measured problem-solving in the way in which this variable was operationalized in our review. Due to the inclusion of problem-solving as part of approach coping, defined as logical analysis, positive reappraisal, seeking guidance and problem solving in McGarrity et al., it is not possible to draw definitive conclusions about the relationship between problem-solving and ED symptoms [29].

## Discussion

The aim of this review was to identify associations between ERS and ED symptoms among both men and women and to indicate sex/gender role in these associations. The data confirmed the relationships between ERS, sex/gender, and ED symptoms, but the specific role of sex/gender in these associations remains unclear.

According to common theoretical models explaining the mechanisms of EDs development and maintenance, dysfunctional or insufficient emotion regulation may lead to eating becoming an escape from poorly tolerated affective states or a substitute regulator [51, 52]. For instance, Fairburn in his transdiagnostic model indicates that mood intolerance (defined as the inability to adequately cope with difficult emotions) may underlie the occurrence of all EDs generally [51]. This systematic review, based on 16 studies, appears to support and complement this model.

This synthesis of the analyzed research proves to be consistent with other observations in the literature, indicating that using maladaptive ERS, such as rumination, suppression, or avoidance, is associated with disordered eating in both female and male populations [12, 17]. The acquired data did not allow for drawing conclusions regarding adaptive ERS (i.e., problem solving, acceptance), but they partially confirmed previous findings suggesting that individuals with ED symptoms tend to present lower levels of cognitive reappraisal usage [53]. However, it is noteworthy that the literature indicates that the relationship between maladaptive ERS and EDs is stronger than between adaptive ERS and EDs [12, 54]. This finding results in a greater number of studies and more pronounced associations observed in this field of research.

### Rumination

Rumination was the most frequently assessed emotion regulation strategy in the examined studies, with its measurement conducted in seven publications. All of them demonstrated a positive correlation between rumination and various forms of eating pathology, such as BED symptoms [35], BN symptoms [38], and overall ED symptoms and concerns [27, 31, 36, 37, 39]. These findings align with the consensus in the literature that the tendency to ruminate is particularly crucial for the risk of developing EDs [12, 16].

In four out of the seven studies, it was additionally found that women exhibited higher levels of overall ED concerns and symptoms compared to men [27, 31, 36, 39]. One study reported no such gender difference in the context of BN symptoms [38], while in the two remaining researches, comparisons between female and male participants were not tested. These results are coherent with the extensive data confirming that eating disorders are generally more prevalent in the female population, particularly within Western cultural contexts [55]. Regarding gender differences in the tendency to ruminate, three of the analyzed studies observed that the women participants engaged in more rumination than men [31, 36, 38], one study found no differences [39], while the remaining studies did not report on this aspect. These results are coherent with the current literature indicating that rumination is more common in a female population [16].

The general conclusions regarding the relationship between rumination, sex/gender, and EDs are ambiguous. It remains unclear whether the rumination-ED relationship differs among women and men. Nonetheless, this review provides substantial evidence supporting the hypothesis that rumination, particularly in its brooding facet, is associated with ED concerns regardless of the gender of the participants. Gordon et al. demonstrated that higher levels of ruminative brooding predicted a greater number of BED symptoms for both men and women [35], while another study indicated that a higher reported level of ED concerns served as a predictor for increased tendencies toward brooding [27]. Other findings indicated that female and male individuals with higher levels of rumination exhibited more BN symptoms if they also demonstrated lower levels of self-control [38]. Moreover, the work of Opwis et al. revealed the mediating role of rumination in the relationship between gender and various manifestations of disordered eating (such as food craving, both objective and subjective BED symptoms, and overall ED symptoms) [31]. These findings suggest that the tendency to ruminate may be a critical predisposing factor in explaining gender differences in the symptomatology of EDs, highlighting cognitive factors as more fundamental than previously recognized. It may be important in the broader context of understanding the differences in EDs prevalence among women and men, extending beyond biological factors and cultural standards of beauty. Consequently, they may have important implications for the planning of therapeutic interventions and prevention strategies. Noteworthy, the relationship between rumination and EDs symptomatology in the female population might indeed be biologically conditioned, as suggested by the findings from the twin study by Johnson et al. [36]. Their research indicated that genetic factors better explain the relationship between rumination and generalized ED symptoms in women compared to men, which may suggest that there are in fact biological variables responsible for both rumination and ED concerns in the female population. However, since only one of the analyzed studies considered genetic and environmental factors, drawing meaningful conclusions necessitates further research in this area.

It is not possible, based on the collected studies, to reliably determine whether and how the strategy of rumination is specifically associated with particular ED symptoms depending on sex/gender. Only one of the included studies presented substantial findings in this context, showing that ruminative brooding was a salient factor contributing to BED frequency in women, but not in men [37]. This supports the assumption that rumination has a different role in the development and course of EDs in the female population, although further exploration is still required.

#### Suppression

Suppression emerged as a significant predictor of EDs. The use of this ER appears to be associated with higher level of EDs. However, it is not clear whether men or women use suppression more frequently in the context of EDs, as study findings are mixed. Some research suggests that women use suppression more often [30], while other studies indicate that, when considering specific symptoms, men may use suppression more [37]. Additionally, some studies report no significant gender differences [43]. Therefore, it appears that suppression plays a role in EDs for both women and men, but the extent and nature of this role require further investigation. An interesting point for future research is that suppression may act as a mediator for certain variables (anxiety, uncertainty intolerance), more precisely described in the results, which offers promising potential for deeper analysis [42, 45]. Future research testing its mediating role may help uncover the mechanisms underlying various psychiatric disorders. While the findings generally align with common observations that thought suppression is maladaptive in the context of disordered eating [12], there is still a need for further research to determine whether it is indeed more meaningful in the male population.

#### Avoidance

Avoidance emerged as a factor significantly related to EDs symptomatology in two of the three studies reviewed [27, 29]. This relation may be bidirectional as McGarrity et al. found that avoidance significantly predicted concerns related to EDs and binge eating behavior, while Rawal et al. demonstrated that greater ED concerns were also predictive of increased avoidance tendencies [27, 29]. In both cases, the observed relationships pertained to both the female and male participants. However one study observed a positive correlation between female gender and higher levels of avoidance [29], other studies did not report significant gender differences in avoidance, nor any interaction effects between gender, avoidance, and ED symptoms [27, 47].

Given these data, it remains unclear whether heightened avoidance leads to an increase in eating disorder symptoms, or whether greater eating disorder concerns intensify avoidance strategies. In this context, the findings of Denning et al. are noteworthy, as they indicate that the strongest predictor of overall ED symptoms was the strategy of seeking reassurance, while avoidance was found to be insignificant [47]. Reassurance seeking, defined as a strategy used to regulate affect by behavior that involves excessive seeking of information and reassurance from others, was not assessed in other studies. However, previous research has shown that this variable positively correlates with avoidance coping and strengthens the association between social avoidance and overall EDs psychopathology in women diagnosed with BN [56]. Therefore, despite substantial empirical evidence supporting the relationship between avoidance and EDs, these findings suggest paths worth exploring in further research. More studies are needed to investigate the role of avoidance in disordered eating while controlling other strategies, e.g. intrapersonal coping. Moreover, additional research will be necessary to clarify the nature of the associations between the avoiding regulation strategy and gender.

#### Reappraisal

Although reappraisal was examined only in five included studies, the data consistently show that reappraisal is linked to lower severity of ED symptoms. Importantly, findings suggest that reappraisal plays a role in BN and BED, as its use may be linked to a reduction in symptom severity. However, this strategy may not play a similar role in OSFED or AN. This may be explained by evidence from Svaldi et al., indicating that individuals with BED tend to have a greater capacity for emotion recognition and effective emotional regulation compared to those with other ED diagnoses [57]. Meanwhile, studies report this association for both women and men [7, 28], while others find that the relationship is stronger among women [7]. Findings from Perchtold et al. on depression suggest that reappraisal is an important emotion regulation strategy for both men and women, but it may be more effective in reducing symptoms among men [58]. In women, its benefits appear to depend more on the interplay with other regulatory strategies. This distinction may provide a valuable basis for future research exploring the role of reappraisal specifically in individuals with EDs.

#### Acceptance

Broader conclusions regarding acceptance cannot be drawn, as only one of the included studies conceptualized acceptance in line with the definition adopted in this review [47]. This highlights a potential issue with the current conceptualization of ERS. Acceptance has been defined in various ways in the literature - sometimes as a rather adaptive one, when considered as non-elaborative, non-judgmental, present-centered awareness in which thoughts, feelings, and sensations are accepted as they are [12]. Other times, when it is considered as acceptance of resignation - part of an avoidance coping strategy, acceptance is viewed as a maladaptive strategy [29]. To promote consistency in how these concepts are defined in future research, it might be beneficial to conceptualize ERS without indicating their adaptive or maladaptive character, which is in line with the emotion regulation flexibility framework.

#### Problem solving

Problem-solving was not directly assessed in any of the included studies as defined in this review. In McGarrity et al., it was incorporated into a broader approach coping construct alongside other strategies such as logical analysis and positive reappraisal [29]. Although this strategy appears to be protective, its conceptualizations vary widely and are inconsistent across studies [29, 59], which hinders precise evaluation of its role in the development and maintenance of symptoms in psychiatric disorders.

## Summary

Considering sex/gender differences in emotional regulation, it is believed that women have a greater tendency to use passive coping strategies, showing a higher propensity for rumination and self-blame [60], which is associated with an increased risk of various disorders, particularly depression, but also EDs [12, 16, 17]. In contrast, males are more inclined to prioritize problem-solving objectives, utilizing reappraisal strategies to manage or modify the circumstances they perceive as influencing their emotional states, rather than seeking to alter their self-perception [60]. Noteworthy, although there is a known tendency for women to be more inclined to employ rumination as a regulative strategy than men, the data presented in this review indicated that rumination is a significant predictor of ED symptoms in both women and men, and may be a stronger predictor than sex/gender. This observation needs further empirical verification, considering the possibility that the increased tendency for rumination in women might be one of the factors explaining why females more frequently develop EDs, while men with a propensity for rumination may be at risk for developing such symptoms. An important role of rumination is supported by previous research, which suggest that the association between rumination and many types of psychopathology tends to be stronger compared to other ERS [12, 16]. Further research is warranted to investigate the relationship between rumination and the symptomatology of EDs, taking into account potential sex/gender differences, particularly highlighting brooding, a subtype of rumination that has already been shown to be a strong risk factor for other disorders, such as depression [61, 62].

The results of this review suggest that the association of using maladaptive ERS with ED symptoms is stronger among women than men. However, this conclusion may be limited by the fact that measures for ED symptoms are often recognized as developed with female-specific presentations in mind [63]. In reviewed studies, the assessment of ED symptoms included mainly measures assessing symptoms such as dietary restraint, eating concerns, and weight/shape concerns or symptoms of AN, BN or BED [64, 65]. These tools have been primarily validated in female samples [4, 66, 67] or groups with a distinct female majority [68]. As mentioned, the symptom profile of EDs in men may differ in terms of experienced symptoms. Future research should more extensively include instruments that assess symptoms more specific to men, for example, concerns about muscularity, with body-shaping behaviors primarily involving intense exercise aimed at enhancing muscularity.

Furthermore, this systematic review indicates not only the need for further exploration of sex/gender differences in ERS, but also the need for ongoing research testing the relationship between specific ERS and ED symptoms while controlling for sex/gender or testing the moderating role of sex/gender in this relationship.

In almost all of the reviewed articles, both ERS and sex/gender were treated as predictors of ED symptoms in the regression analyses. However, no interactions between ERS and sex/gender were tested, and thus, verifying the moderating role of sex/gender in the relationship between ERS and ED symptoms was impossible [69]. An alternative approach could involve studies comparing groups of women and men, but such analyses would require adequately large subgroups in clinical populations and controlling for confounding variables [70]. Mediation analysis was conducted in only one of the included studies, which revealed that rumination mediated the relationship between gender and ED symptoms [31].

Moreover, previous studies have demonstrated that individuals with BED provided a less favorable evaluation of their ability to influence their emotional states compared to those with AN, BN and OSFED [71]. Therefore, it might also be beneficial to control for specific ED diagnoses. This review suggests that certain ERS may be specifically associated with particular symptom clusters, and this effect may be sex/gender-dependent. For instance, Mitchell et al. demonstrated that in BN group, the strategy of reappraisal was a significant predictor of ED symptoms while controlling for sex, whereas in OSFED group, it was the suppression strategy that proved to be a significant predictor [44]. Similarly, in studies by Rosenbaum and White, it was found that anxiety was associated with BED symptoms indirectly through suppression, but this effect was observed only in female group [42]. These interesting observations indicate the need for further research considering potential differences between specific clinical groups of men and women.

## Limitations

A common limitation of the research included in the review, despite the substantial evidence linking emotion regulation (ER) with various forms of psychopathology, including EDs, is the use of a cross-sectional design [72]. Given the lack of experimental or even longitudinal studies, it is not possible to determine whether the observed deficits in ER are a basis for the development of EDs, or whether they result from symptoms, a distorted body image, and eating habits. This difficulty in ER studies concerning other forms of psychopathology has already been highlighted by other researchers [73]. Moreover, the results of mediation analysis performed in included studies are of less impact when using a cross-sectional design, as such a design does not allow for determining the temporal sequence between the variables involved in the mediation analyses [74]. Therefore, to thoroughly understand this issue, alternative research designs including measuring ERS and ED symptoms over time, will be essential.

Another limitation across studies is the lack of specifying whether biological sex or psychological gender was measured. Although some researchers acknowledge the distinction, data from transgender and gender-diverse individuals are often excluded. This raises ethical and methodological concerns, as excluding these groups may bias estimates of the true prevalence of psychiatric health issues, including EDs [75].

Our review highlights the potential importance of sex/gender in the relationships between ERS and EDs, although it does not allow for a definitive conclusion about the exact nature of this role. This is because only one of the incuded studies examined sex/gender in interaction with ERS, providing a more in-depth response to one of our research questions. The remaining studies addressed sex/gender in different ways: seven tested it as a predictor, four as a covariate, and one as a correlate. Additionally, three studies reported separate analyses for women and men. Taken together, these findings suggest promising directions for future research, particularly the need for studies that explicitly examine the potential moderating role of sex/gender in the association between ERS and EDs in order to address the existing gaps in literature.

Research analyzed in this review has primarily been conducted in the USA, which does not necessarily limit the generalizability of the findings, as there is evidence indicating that the prevalence of EDs, according to the DSM-5 classification, might be similar across other continents [76]. However, the DSM-5 criteria primarily pertain to the clinical manifestation of EDs in women, making it difficult to determine these differences in the context of men [77].

This study includes only scientific publications whose full text was in English. Additionally, the search string primarily included key terms related to EDs and ERS, without the use of truncations. Due to this, there is a possibility that an article meeting the inclusion criteria and potentially offering additional insights into the topic may have been unintentionally missed.

Summing up, the findings of this systematic review revealed a elementary lack of data regarding the role of sex/gender in the association between specific ERS and ED symptoms. Yet, the review indicated that different ERS may be used by women and men with specific EDs. These conclusions are crucial for future research, as their outcomes could directly impact the effectiveness of preventive and therapeutic interventions.

## Supplementary Information

Below is the link to the electronic supplementary material.

Supplementary Material 1.

## Data Availability

No datasets were generated or analysed during the current study.
